# Facile Preparation of Micrometer KClO_4_/Zr Energetic Composite Particles with Enhanced Light Radiation

**DOI:** 10.3390/ma12020199

**Published:** 2019-01-09

**Authors:** Xiaoli Kang, Chunhong Li, Zhou Zheng, Xudong Cui

**Affiliations:** 1 Institute of Chemical Materials, China Academy of Engineering Physics, Mianyang 621900, China; zhou_chen1118@126.com (Z.Z.); xudcui@caep.cn (X.C.); 2 School of Materials Science and Engineering, Xihua University, Chengdu 610039, China

**Keywords:** Potassium Perchlorate/Zirconium (KClO_4_/Zr) composite, light radiation, frame photographs, reaction pathways

## Abstract

Developing energetic composite materials consisting of fuel and oxidizer is an effective strategy to enhance the energy release property. However, this strategy has rarely been applied in Potassium Perchlorate (KClO_4_)-containing energetic materials, even though KClO_4_ is a much stronger oxidizer than most previously reported metal-oxide oxidizer. One of the main obstacles is the lack of simple and in situ ways to introduce KClO_4_ into the composite. In present work, micrometer KClO_4_/Zirconium (KClO_4_/Zr) composite particles were successfully prepared using a facile chemical solution-deposition method. The structure and particle morphologies of as-obtained KClO_4_/Zr composite were characterized by X-ray diffraction (XRD) and scanning electronic microscope (SEM)-EDS (Energy Dispersive Spectrometer). The evolutionary combustion behavior was evaluated using flame-based light-radiation spectra and successive photography technique. Results showed that the morphology, light-radiation properties and flame-evolution characteristics of KClO_4_/Zr composite varied with the content of KClO_4_ and the particle size of Zr. Compared with the mechanical mixture of KClO_4_/Zr, the KClO_4_/Zr composite showed much higher light-radiation intensity and longer light-emission duration time after reasonably controlling the preparation parameters. Flame photographs revealed that the enhanced light radiation of KClO_4_/Zr composite should be ascribed to higher use efficiency of “oxygen” in the oxidizer, which promoted both the solid–solid and solid–gas reaction pathways between KClO_4_ and Zr.

## 1. Introduction

Pyrotechnics are kinds of energetic materials which can produce specific effects of light, heat, sound, and smoke, etc., having wide applications in military and civil fields [1,2,3,4]. Typically, pyrotechnic compositions are composed of mixtures of oxidizer and fuel. One of the important parameters influencing the reaction and properties of pyrotechnic compositions is the contact intimacy between oxidizer and fuel. Since a lot of pyrotechnic reactions involve the solid–solid and solid–gas reaction pathways, close contacting of participating particles will promote the mass and heat transfer during reactions, so that the final combustion property can be enhanced. Except for developing effective mixing method and technology, preparing composite materials is viewed as an ideal solution to obtain high-performance pyrotechnics [5]. By intelligently designing the structure of the composite, the contact intimacy between oxidizer and fuel, and thus the energy release property, can be greatly improved [6].

So far, depending on the characteristics of oxidizer and fuel, there have been a lot of methods such as thermal evaporation [7], magnetron sputtering [8], sol-gel [9,10], atomic layer deposition [11,12], electrospray assembly [13], electrophoretic deposition [14], and electrospinning [15] etc. to prepare pyrotechnic composites. However, most of the oxidizers involved in previous energetic composites were metal-oxide; while strong oxidizers such as the commonly used perchlorate salt (KClO_4_, NaClO_4_, etc.) in pyrotechnics were scarcely reported [13,16,17,18] due to the lack of simple and in situ ways to produce these perchlorate salts in the composite. Another problem is that most of the previously reported pyrotechnic composite was based on at least one kind of nano-scale raw materials such as nanometer particles and nanowires, while micro-scale raw materials were rarely used [13]. It is well known that the self-oxidization for nanometer metal fuels is often more serious than that for the micrometer fuels, which will cause undesirable energy loss. Besides, nanometer components are easily to agglomerate during processing due to their high surface energy. If micro-scale fuels can be introduced into energetic composite to replace corresponding nanometer fuels, the energy density (J/g), processing safety, storage stability and cost of the energetic composites will be improved.

In the present work, a facile method without the use of any assistant agents, harmful solvents, high temperature and complex experimental devices was developed to prepare micrometer KClO_4_/Zr pyrotechnic composite, which was a typical flash composition [19]. The core idea was that the oxidizer KClO_4_ was chemically deposited on surface of commercial micrometer Zr particles through double-decomposition reaction. The phase composition, morphology, light-radiation property and flame photographs of as-obtained KClO_4_/Zr composite were characterized. The obtained micrometer KClO_4_/Zr composites were demonstrated to have significantly enhanced light-radiation property than that of their corresponding mechanical mixtures.

## 2. Materials and Methods

KClO_4_/Zr composite was prepared by chemically deposited KClO_4_ on surface of micrometer Zr particles via double-decomposition method. The double-decomposition reaction could be described as follows:

(1) $${\mathrm{NH}}_{4}\mathrm{Cl}O_{4}+\mathrm{KOH}=\mathrm{KCl}O_{4}+NH_{3}↑+H_{2}O$$

**Figure 1:**
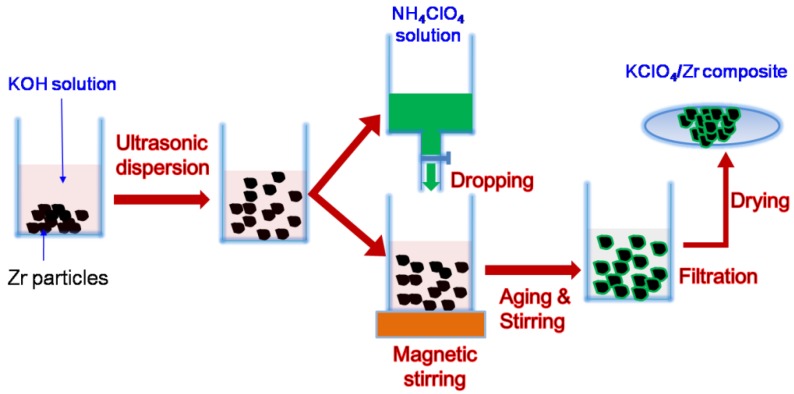
Schematic diagram illustrating preparation process of KClO_4_/Zr composite.

The schematic diagram illustrating preparation process was presented in Figure 1. NH_4_ClO_4_ and KOH were of analytical grade and used without treatment. Three kinds of micrometer Zr powders with averaged particle sizes of 3 μm (Shanghai St-Nano Science and Technology Co., Ltd., Shanghai, China), 6 μm (Jinzhou Institute of Metal Material, Jinzhou, China) and mesh 400 (General Research Institute for Nonferrous Metals, Beijing, China) were used, respectively. In a representative experiment, 0.8 g KOH and 0.8 g Zr powder were added into 30 mL de-ionized water, and the suspension was ultrasonically oscillated for 15 min to break the conglomeration of Zr particles. Then the suspension was magnetically stirred to avoid sedimentation of Zr particles. 1.2 g NH_4_ClO_4_ was dissolved into 20 mL de-ionized water, and the obtained NH_4_ClO_4_ solution was dropped into the aforementioned suspension by an injection pump with a rate of <0.4 mL/min. During dropping, the suspension was stirred at the rate of >550 r/min. After dropping, the suspension was stirred for another 60 min with the same stirring rate. Finally, particles in the suspension were separated out through vacuum filtration and dried at 60 °C in a vacuum oven. The averaged pore size of the filter paper was 1~3 μm. All the manipulations were carried out under room temperature. The room temperature in this study was controlled to be 21 °C with an air conditioner. The mass ratio of KClO_4_ to Zr can be easily adjusted by changing the usage of Zr powders, concentration of reactants solution, reaction time and temperature.

Mechanical mixture of KClO_4_/Zr was used as reference samples. In reference samples, the averaged particle size of KClO_4_ was 6 μm, and the mass percent of KClO_4_ was the same as that in the KClO_4_/Zr composite. KClO_4_/Zr mixture was prepared by manually mixing KClO_4_ powders with Zr powders using an agate pestle and motor.

Morphology and phase composition of the obtained KClO_4_/Zr composite were characterized by scanning electronic microscope (SEM) (FEI, Portland, USA) and X-ray diffraction (XRD) (Bruker, Karlsruhe, Germany), respectively. The mass ratio of KClO_4_ to Zr in the composite was quantified by measuring the content of potassium element via ICP (inductively coupled plasma) technique. Light-radiation property of combusting KClO_4_/Zr was characterized by flame spectra, temporal evolution of light intensity and high-speed photography measurement. Flame spectra were collected by a fiber-optic spectrometer (425 nm~950 nm, Avantes 2048, Avantes, Apeldoorn, The Netherlands). Evolution of light intensity with time was monitored by a silicon photodiode (DET10 A, 200 nm~1100 nm, Thorlabs, Newton, NJ, USA) combined with a Tektronix oscilloscope (DPO 4054 B, Tekttronix, Beaverton, OR, USA). For this set of experiments, 20 mg powder samples were put on the sample pan and ignited by a hot wire with a DC power. The occupying area of each sample on the sample pan was fixed to the same (10 mm × 1.5 mm, length × width). The hotwire (0.5 mm in diameter) was embedded inside the sample powders. The detectors of the fiber-optic spectrometer and the silicon photodiode were set on two sides of the sample from a certain distance, respectively, and faced the sample in the perpendicular orientation (The schematic diagram of the experimental systems could be seen in Figure S1 in the Supplementary Materials). Flame images sequence was recorded with a high-speed camera (Phantom MIRO R310, Ametek, Wayne, NJ, USA). Sampling rate and exposure time was 2000 f/s and 100 µs, respectively. In this test, 25 mg powders were put on the sample pan and ignited by a hotwire with a DC power.

## 3. Results

### 3.1. Sample Characterization

**Figure 2:**
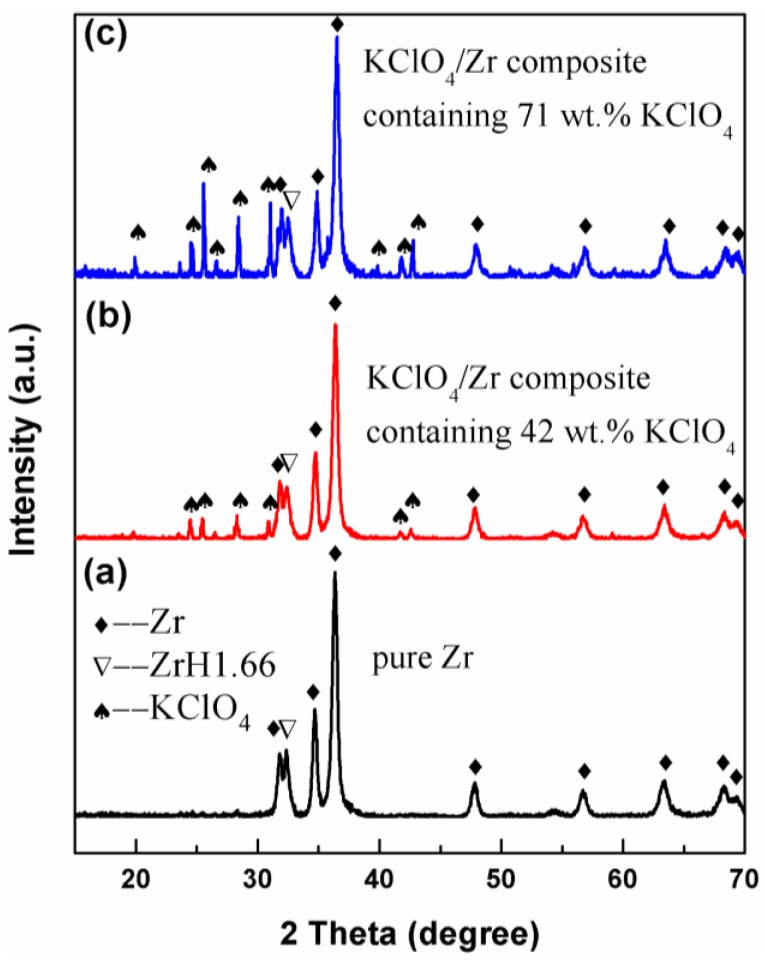
XRD patterns of (**a**) pure Zr (3 μm), (**b**) KClO_4_/Zr (3 μm) composite containing 42 wt.% KClO_4_ and (**c**) KClO_4_/Zr (3 μm) composite containing 71 wt.% KClO_4_.

Figure 2 showed comparison of XRD patterns of pure Zr powders (3 μm) with as-prepared KClO_4_/Zr composite samples prepared under different experimental conditions. Figure 2a showed that XRD pattern of raw Zr powders was composed of Zr peaks and small amount of ZrH_1.66_ peak. The main diffraction peaks of KClO_4_/Zr composite samples (Figure 2b,c) were identified to be KClO_4_, Zr, and ZrH_1.66_. This result directly confirmed the existence of KClO_4_ in the composite. Also, it was suggested that the double-decomposition reaction was completed, and no unreacted reactants were detected in the final composite.

**Figure 3:**
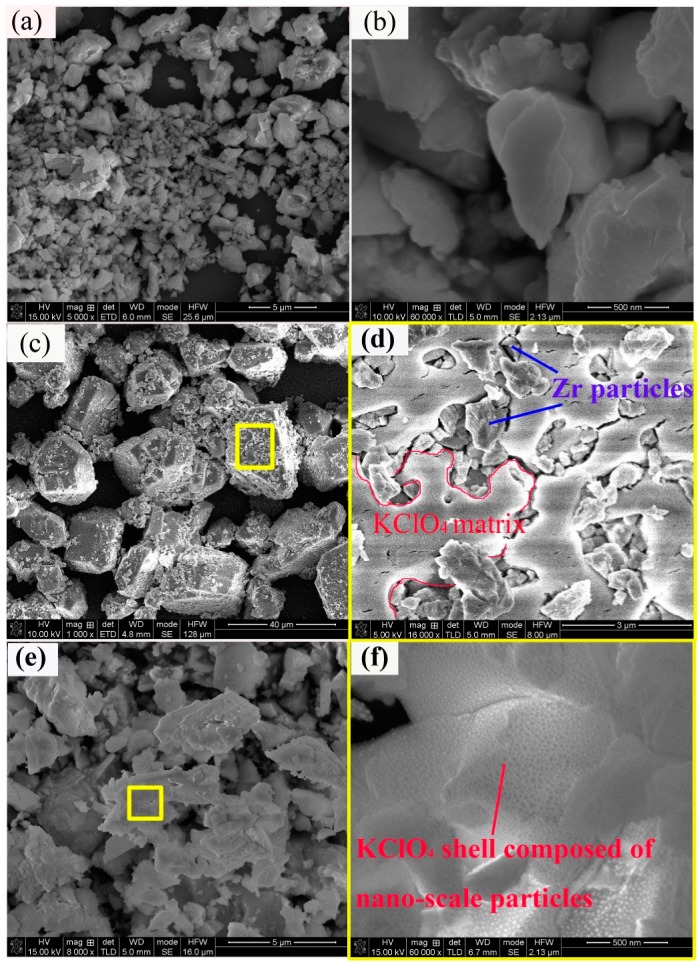
Surface morphology of 3 μm Zr powders (**a**), smooth surface of Zr particles (**b**); as-prepared KClO_4_/Zr (3 μm) (71/29, wt.%) composite (**c**), “embedded type” microstructure of selected area in Figure 3c with Zr particles embedded into consistent KClO_4_ matrix (**d**), as-prepared KClO_4_/Zr (3 μm) (38/62, wt.%) composite (**e**) thin KClO_4_ shell in Figure 3e composed of nanometer particles (**f**).

**Figure 4:**
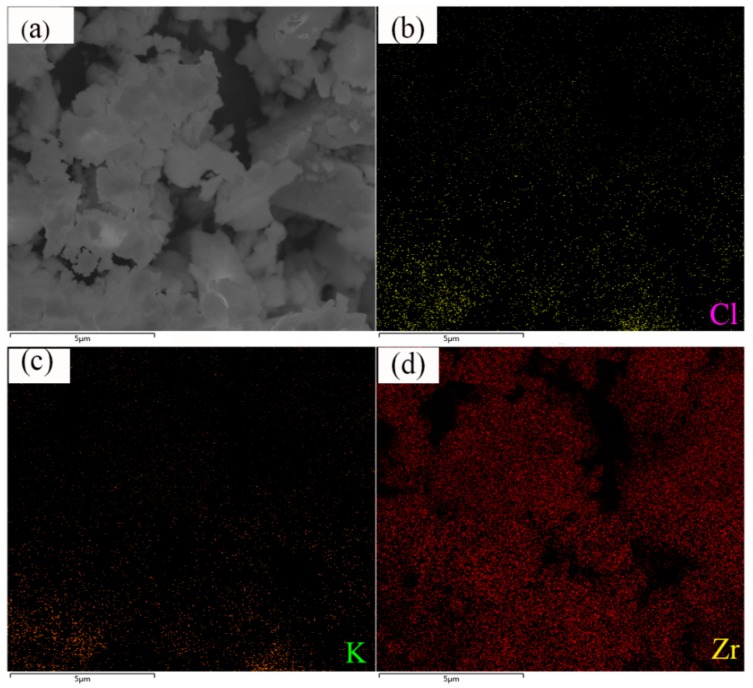
SEM image (**a**) and EDS mapping of Cl (**b**), K(**c**) and Zr (**d**) elements for KClO_4_/Zr (3 μm) (38/62, wt.%) composite.

Figure 3 showed SEM images of raw 3 μm Zr powders and as-prepared KClO_4_/Zr (3 μm) composite samples containing different contents of KClO_4_. It was seen from Figure 3 that raw 3 μm Zr particles were irregular, with particle sizes ranging from hundreds of nanometer to ~several micrometers, and the particle surface was smooth. The morphology of as-prepared KClO_4_/Zr (3 μm) composites (Figure 3c–f) varied with the content of KClO_4_ in the composite. The composite containing high content of KClO_4_ (71 wt.%, Figure 3c) showed very large particles of 20 μm~40 μm. The magnified image (Figure 3d) indicated that each large particle was composed of multiple small Zr particles embedded inside the consistent matrix of KClO_4_ (here we call this structure as “embedded type”). However, the composite containing low content of KClO_4_ (38 wt.%, Figure 3e) showed similar morphology with that of raw Zr particles, except that the particle size became larger, suggesting the KClO_4_ shell was thin. The magnified image (Figure 3f) indicted that the surface of KClO_4_/Zr composite was composed of a lot of nanometer particles. The element mapping of Cl, K and Zr demonstrated the existence and uniform distribution of KClO_4_ on the surface (Figure 4).

**Figure 5:**
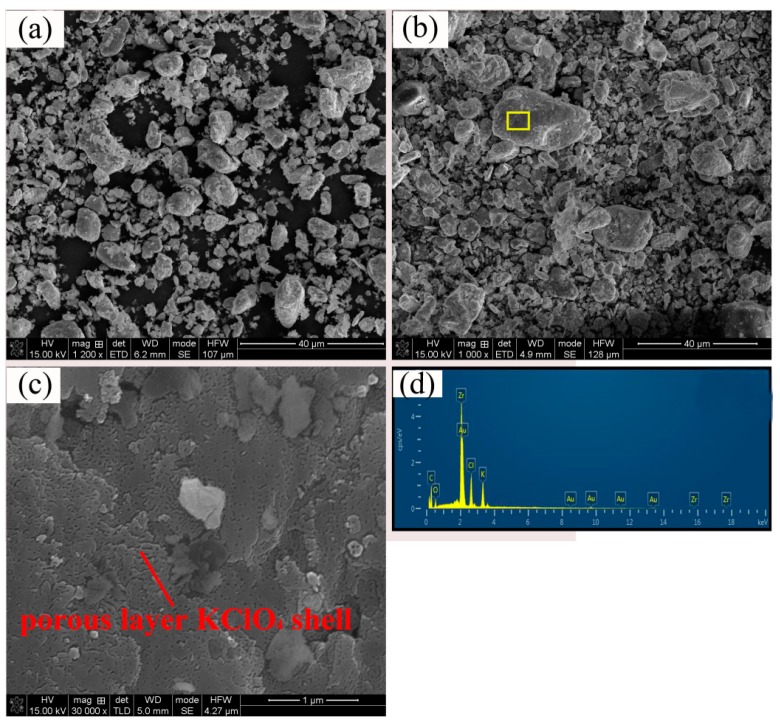
Surface morphology of 6 μm Zr particles (**a**), as-prepared KClO_4_/Zr (6 μm) composite (42/58, wt.%) (**b**), porous layer structure of KClO_4_ shell in selected area(**c**) EDS result of the selected area (**d**).

Figure 5a–c showed SEM images of raw 6 μm Zr powder and as-prepared KClO_4_/Zr (6 μm) composite containing 42% KClO_4_. Due to the thin KClO_4_ shell, the morphology of KClO_4_/Zr (6 μm) composite (Figure 5b) was very identical to the raw Zr particles (Figure 5a), which was similar to Figure 3e. However, the enlarged image (Figure 5c) showed that the surface of KClO_4_/Zr (6 μm) composite had a porous layer structure, and the EDS result (Figure 5d) demonstrated the existence of KClO_4_ on the surface of selected area in Figure 5c. It was found that when the particle sizes of Zr were further increased to mesh 400, homogeneous KClO_4_/Zr (mesh 400) composite could not be obtained (Figure S2). Reasons may be that the particle size distribution of mesh 400 Zr powders was relative wide, ranging from several hundred nanometers to several ten micrometers, resulting in distinct differences in the surface activity of Zr particles. During the nucleation process of KClO_4_, only those small Zr particles were spontaneously selected as the crystal nucleus to form KClO_4_/Zr composite, while those large Zr particles were left alone. Simultaneously, the homogeneous nucleation and growth of KClO_4_ occurred. The finally obtained products were in fact the mixture of KClO_4_, Zr (mesh 400) and small amount of KClO_4_/Zr composite, which were not suitable for application due to the poor controllability in preparation.

The above-mentioned morphology change of deposited KClO_4_ was interpreted as follows. The deposition process of KClO_4_ shell on the surface of Zr particles included several steps. (1) K^+^ ions in the KOH solution adsorbed on the surface of Zr particles. (2) Negative ClO_4_^−^ ions in the dropped NH_4_ClO_4_ solution bound with the positive K^+^ ions on the surface of Zr particles, producing KClO_4_ crystal nucleus. (3) The adsorption process of K^+^ ions and the binding process of K^+^ ions with ClO_4_^−^ ions circulated alternatively, resulting in the growth of KClO_4_ on the surface of Zr particles. The morphology of KClO_4_ was determined by the initial amount and distribution of adsorbed K^+^ ions on the surface of Zr particles, which determined the site and distribution of initial KClO_4_ crystal nucleus, and thus the subsequent crystal growth. Therefore, the surface characteristics of raw Zr particles were the key. The sites available for K^+^ ions adsorption on the surface of 3 μm Zr particles should be dispersive, so the nucleation and growth of KClO_4_ displayed an island-mode, resulting in a KClO_4_ shell composed of small isolated particles (like the results in Figure 3e,f). However, this circumstance changed when the content of Zr particles in the suspension was very low (like the results in Figure 3c,d). In this case, the self-nucleation and growth process of KClO_4_ from the solution proceeded simultaneously with the heterogeneous nucleation on the surface of Zr particles, producing very large “embedded type” KClO_4_/Zr composites. In contrast, the number of sites available for K^+^ ions adsorption on the surface of 6 μm Zr particles should be huge, so the nucleation of KClO_4_ occurred uniformly across the whole surface of Zr particles, and the growth of KClO_4_ displayed a layered mode. The porous structure was caused by the escape of gas products of NH_3_ (like the results in Figure 5).

### 3.2. Light-Radiation Property

**Figure 6:**
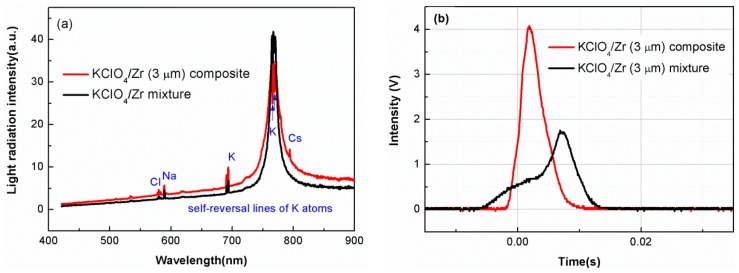
Flame spectra (**a**) and light-emission traces (**b**) for KClO_4_/Zr (3 μm) composite (red line) and KClO_4_/Zr (3 μm) mechanical mixture (black line).

Since the KClO_4_/Zr composite having high content of KClO_4_ (higher than 60%) could not be ignited or could not keep stable combustion, only the near-stoichiometric composites were selected to test the light-radiation property. Figure 6a,b showed comparison of flame spectra and light-emission traces of KClO_4_/Zr (3 μm) composite and corresponding mechanical mixture, respectively.

The spectra in Figure 6a were dominated by the continuous background, over which were superposed some selective emission bands of neutral K, Cl, and impurities of Na and Cs [20]. It was seen from Figure 6a that light-radiation intensities at almost all wavelengths except the local intensity decrease around 766 nm for the KClO_4_/Zr composite sample were stronger than that of the mechanical mixture. This local intensity decrease around 766 nm was originated from the self-reversal of K atoms in the combusting flame. The self-reversal behavior may fluctuate depending on several complex factors such as the K atoms concentration and temperature gradient of flame, but it did not influence the increasing trend of total light-radiation energy within the whole wavelengths. The total light-radiation energy could be relatively compared by the integrated area of the spectra curves in the wavelength range of 420~900 nm. The calculated value of integrated area for KClO_4_/Zr composite was 32% larger than that of mechanical mixture (3145 vs. 2375), indicating higher light-radiation energy. Figure 6b revealed that the peak intensity of light-emission trace for KClO_4_/Zr composite was more than two times of its counterpart, indicating higher peak light-radiation power.

**Figure 7:**
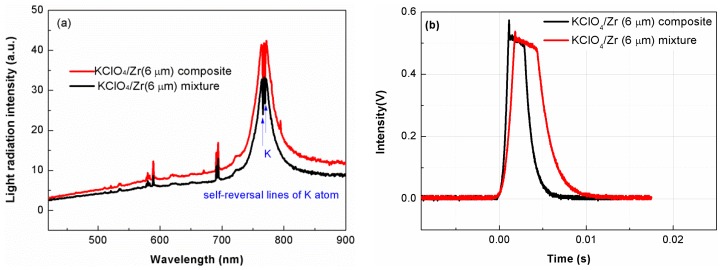
Flame spectra (**a**) and light-emission traces (**b**) for KClO_4_/Zr (6 μm) composite (red line) and KClO_4_/Zr (6 μm) mechanical mixture (black line).

Figure 7 showed comparison of flame spectra and light-emission traces of as-prepared KClO_4_/Zr (6 μm) composite and corresponding mechanical mixture, respectively. Figure 7a demonstrated the KClO_4_/Zr (6 μm) composite had obviously higher light-radiation intensities at all wavelengths, indicating enhanced light-radiation energy. Different from the results in Figure 6b, light-emission traces in Figure 7b indicated that the peak radiation intensity of the KClO_4_/Zr (6 μm) composite did not show an increase, but the duration time near peak emission intensity increased by ~30%.

The enhanced light-radiation properties for composite samples should be ascribed to two aspects. The first was the increased contact intimacy between Zr and KClO_4_ by formation of core-shell structure, which shortened the distance for heat and mass transfer, and promoted the proceeding of exothermic reaction. The second was the particle fining of KClO_4_ component in the composite. As had been shown in SEM images (Figure 3 and Figure 5), KClO_4_ in the composite sample had a nano-scale particle or porous layer structure, which was smaller than the ~6 μm particles used in the mechanical mixture samples. These results also revealed that by artificially controlling the preparation parameters and the structure of KClO_4_/Zr composite, its light-radiation property could be finely tuned, for example, one could tailor the composite to meet the requirement of higher peak radiation intensity (as shown by red line in Figure 6b) or longer duration time near peak radiation (as shown by red line in Figure 7b).

### 3.3. Flame Photographs

**Figure 8:**
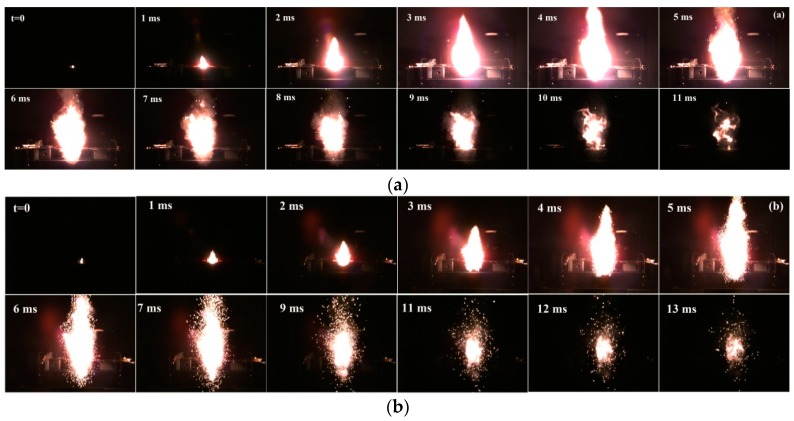
Flame sequence of combusting KClO_4_/Zr (3 μm) composite (**a**) and KClO_4_/Zr (3 μm) mechanical mixture (**b**).

**Figure 9:**
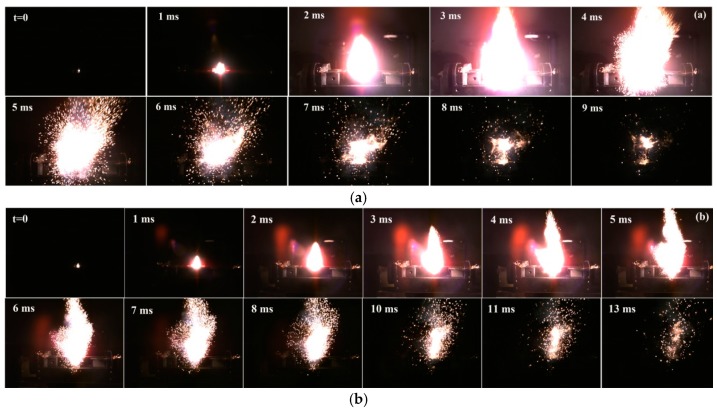
Flame sequence of combusting KClO_4_/Zr (6 μm) composite (**a**) and KClO_4_/Zr (6 μm) mechanical mixture (**b**).

Figure 8 and Figure 9 showed the flame sequences of KClO_4_/Zr (3 μm) composite, KClO_4_/Zr (6 μm) composite and corresponding KClO_4_/Zr mechanical mixtures. It was necessary to point out those images for composite samples in Figure 8b and Figure 9b were captured from a farther distance than the corresponding mechanical mixture samples in Figure 8a and Figure 9a, because the surface areas of flames were too larger than the field view of camera. Thus, these images were only used to qualitatively compare their combustion and light-radiation variation characteristics with the reference samples, trying to find clues on different reaction mechanisms.

It was seen that all samples underwent ignition, flame propagation and decay processes accompanied by the variation of light-radiation intensity. Generally, ignition started from the local hot point, leading to exothermic reaction between KClO_4_ and Zr. The released heat energy transferred to adjacent zones, resulting in enlargement of the reaction area until nearly all the samples in the pan began to combust. At this time, surface area and light-radiation intensity of the flame both reached the peak, as could be characterized by the images at 4 ms and 5 ms in Figure 8a,b, and the images at 3 ms and 5 ms in Figure 9a,b, respectively. During this stage, strong red-light halo could be seen around the rim of the flame. By referencing the flame spectra in Figure 6a, Figure 7a and our previous work [20], it was suggested that the observed red-light halo was mainly originated from the emission of gaseous K atoms in the flame around 766 nm. After this peak moment, the red-light halo weakened rapidly, and a lot of particles spattered out of the flame surface. This should be resulted from the expansion of the gaseous products in the high temperature flame. Simultaneously, the light-radiation intensity also decreased rapidly. In this stage, combustion process should be dominated by the condensed phase reactions.

The difference between composite samples and physic mixture samples included two aspects. Firstly, the flame propagation rate for KClO_4_/Zr mechanical mixture was higher than the composite sample, as could be represented by the time consumed from the ignition to the time when flame surface area reached the maximum. Secondly, the duration time of the condense phase combustion after the gas expansion stage for the composite sample was longer than the mechanical mixture sample. Therefore, the light radiation kept longer time at high intensities. This was benefit for the applications where both strong light-radiation intensity and long duration time were pursued.

These differences revealed that reaction mechanism for the KClO_4_/Zr composite was different from that of the mechanical mixture. It was known that the reaction between KClO_4_ and Zr was a multi-phase reaction process and at least included the following four sub-reactions [21,22,23,24]:

(2) $$\mathrm{KCl}O_{4}+2\mathrm{Zr}\to \mathrm{KCl}+2\mathrm{Zr}O_{2}$$

(3) $$\mathrm{KCl}O_{4}\to \mathrm{KCl}O_{3}+O$$

(4) $$\mathrm{KCl}O_{4}\to \mathrm{KCl}+2O_{2}↑$$

(5) $$\mathrm{Zr}+O_{2}\to \mathrm{Zr}O_{2}$$

Equation (2) represented the reaction between solid Zr and solid KClO_4_. Equations (3)–(5) descripted the reaction between solid Zr and gas oxygen released by decomposition of KClO_4_. So far, the detailed reaction mechanisms on the specific role of solid–solid reaction and solid–gas reaction in the ignition and combustion process of KClO_4_/Zr composition were still not clear. References [21,22] pointed out that solid–solid reaction between KClO_4_ and Zr occurred before the solid–gas reaction between Zr and the released gas oxygen, since the exothermic temperature of KClO_4_/Zr mixture was lower than the thermal decomposition temperature of KClO_4_. SEM images in Reference [25] also showed that the solid–solid reaction between KClO_4_ and Zr occurred at low temperatures of 300~500 °C, but TG curves showed obvious weight loss when the temperature was above 400 °C, indicating that with the increase of temperature, the reaction between solid Zr and gas oxygen became dominant.

Based on these viewpoints, the faster propagation rate and expansion of flame for the KClO_4_/Zr mechanical mixture in this work should be ascribed to the higher content of gas oxygen in the flame. That is, solid–gas reaction dominated in the reaction of KClO_4_/Zr mechanical mixture. The gas oxygen was originated from the decomposition of KClO_4_. Due to the limited contact intimacy between reactants (i.e., KClO_4_ and Zr) in the mixture and the restriction of reaction kinetics, the consumption rate of the gas oxygen was slower than its producing rate [26], so some of them unavoidably escaped outward from the reaction system, causing fast expansion of the flame. The role of gas product release on the flame propagation velocity of other energetic compositions has been explained in previous studies [27,28,29]. While for the KClO_4_/Zr composite, the contact intimacy between KClO_4_ and Zr was improved, so the solid–solid reaction may surpass the solid–gas reaction in the initial combustion, when the temperature of the reaction area was still low, inducing less decomposition of KClO_4_ and less oxygen release. On the other side, when the flame temperature was high enough and the solid–gas reaction between Zr and the released oxygen became dominant, the released gas oxygen would easily react with Zr rather than escape out of the flame surface because of the intimate contact between KClO_4_ and Zr. These two effects both resulted in the decrease of gas oxygen in the flame of KClO_4_/Zr composite samples, and thus explained the slow flame propagation rate for the composite samples. However, this was not a bad thing, because more “oxygen” in the oxidizer KClO_4_ was used to complete the combustion reaction, which produced higher combustion heat and enhanced light-radiation property of the KClO_4_/Zr composite. In contrast, though the flame propagation rate was faster for the KClO_4_/Zr mechanical mixture due to the large amount of gas oxygen in the flame, this caused the sacrifice of some reaction heat, as some of the unreacted Zr particles spattered out of the flame surface except for the escape of gas oxygen. This loss of combustion heat was probably the main reason for the less light-radiation energy released by the mechanical mixture samples.

## 4. Conclusions

The chemical solution-deposition method based on double-decomposition between KOH and NH_4_ClO_4_ was demonstrated to be an effective way to prepare KClO_4_/Zr composite. One of the key factors influencing homogeneity of the composite was the dimensional uniformity of Zr particles. As-prepared KClO_4_/Zr composite showed different structures depending on the content of KClO_4_. Typically, when the content of KClO_4_ in the composite was high (>60 wt.%), the KClO_4_/Zr composite particle tended to have an “embedded structure” where multiple Zr particles embedded in consistent KClO_4_ matrix. Otherwise, the KClO_4_/Zr composite particle had a core-shell structure composed of Zr cores surrounded by KClO_4_. Also, morphologies of the KClO_4_ shell differed with particle sizes of Zr. For ~3 μm Zr particles, KClO_4_ shell was composed of nanometer particles. For ~6 μm Zr particles, KClO_4_ shell had a porous layer structure. KClO_4_/Zr composites were found to have enhanced light-radiation properties including much higher light-radiation energy/power and longer light-radiation duration time. Frame photographs revealed that the enhanced light radiation of KClO_4_/Zr composite should be ascribed to higher use efficiency of “oxygen” in the oxidizer, which promoted both the solid–solid and solid–gas reaction pathways between KClO_4_ and Zr.

## Supplementary Materials

The following ar33.e available online at http://www.mdpi.com/1996-1944/12/2/199/s1, Figure S1: Schematic diagram of the experimental system for acquisition of flame spectra and light-emission traces. Figure S2: Morphology of raw Zr particles (mesh 400) (a) and as-prepared KClO_4_/Zr (mesh 400) composite with inhomogeneous composition and structure (b).
