# Supplementary material for: Facile Preparation of Micrometer KClO4/Zr Energetic Composite Particles with Enhanced Light Radiation
Source: Materials (Basel). 2019 Jan 9;12(2):199. doi: 10.3390/ma12020199 (PMC6356741; doi:10.3390/ma12020199)
Supplement: Supplementary file 1 [file materials-12-00199-s001.pdf]

materials

Supplementary

Facile Preparation of Micrometer $KClO_4$/Zr Energetic Composite Particles with Enhanced Light Radiation

Xiaoli Kang [1,*], Chunhong Li [2,*], Zhou Zheng [1] and Xudong Cui [1]

[1] Institute of Chemical Materials, China Academy of Engineering Physics, Mianyang 621900, China; zhou_chen1118@126.com (Z.Z.); xudcui@caep.cn (X.C.)
[2] School of Materials Science and Engineering, Xihua University, Chengdu 610039, China
* Correspondence: kangxiaoli@caep.cn (X.K.); lichunhong@ustb.edu.cn (C.L.); Tel.: +86-028-6572-6204 (X.K.)

Received: 2 December 2018; Accepted: 2 January 2019; Published: date

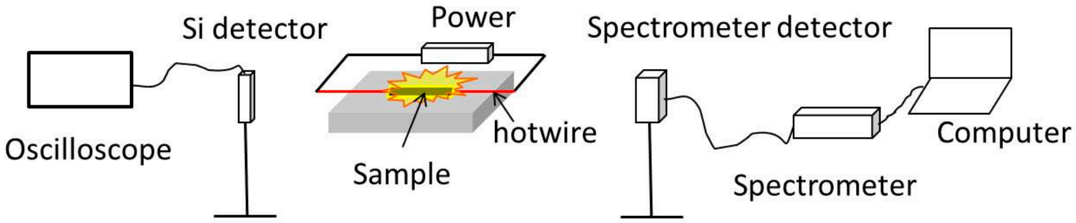

Figure S1 Schematic diagram of the experimental system for acquisition of flame spectra and light emission traces.

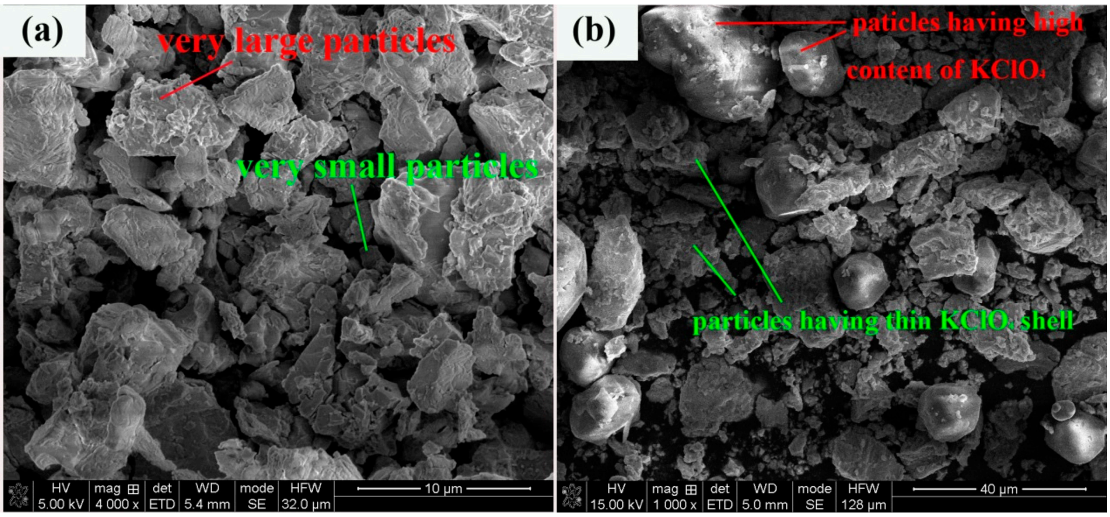

Figure S2 Morphology of mesh 400 Zr powders (a) and as-prepared $KClO_4$/Zr (mesh 400) composite with inhomogeneous structure.
